# Ultrastructural analysis of the decellularized cornea after interlamellar keratoplasty and microkeratome-assisted anterior lamellar keratoplasty in a rabbit model

**DOI:** 10.1038/srep27734

**Published:** 2016-06-13

**Authors:** Yoshihide Hashimoto, Shinya Hattori, Shuji Sasaki, Takako Honda, Tsuyoshi Kimura, Seiichi Funamoto, Hisatoshi Kobayashi, Akio Kishida

**Affiliations:** 1 Institute of Biomaterials and Bioengineering, Tokyo Medical and Dental University, Tokyo, Japan; 2 World Premier International Research Center Initiative, International Center for Materials Nanoarchitectonics (WPI-MANA), National Institute for Materials Science, Ibaraki, Japan; 3 Department of Ophthalmology, Tokyo Medical and Dental University, Tokyo, Japan

## Abstract

The decellularized cornea has received considerable attention for use as an artificial cornea. The decellularized cornea is free from cellular components and other immunogens, but maintains the integrity of the extracellular matrix. However, the ultrastructure of the decellularized cornea has yet to be demonstrated in detail. We investigated the influence of high hydrostatic pressure (HHP) on the decellularization of the corneal ultrastructure and its involvement in transparency, and assessed the *in vivo* behaviour of the decellularized cornea using two animal transplantation models, in relation to remodelling of collagen fibrils. Decellularized corneas were prepared by the HHP method. The decellularized corneas were executed by haematoxylin and eosin and Masson’s trichrome staining to demonstrate the complete removal of corneal cells. Transmission electron microscopy revealed that the ultrastructure of the decellularized cornea prepared by the HHP method was better maintained than that of the decellularized cornea prepared by the detergent method. The decellularized cornea after interlamellar keratoplasty and microkeratome-assisted anterior lamellar keratoplasty using a rabbit model was stable and remained transparent without ultrastructural alterations. We conclude that the superior properties of the decellularized cornea prepared by the HHP method were attributed to the preservation of the corneal ultrastructure.

A worldwide shortage of donated human corneas for transplantation is a serious problem, with increasing need of approximately 1–2 million individuals annually^1^,^2^,^3^. To date, numerous studies have reported various artificial cornea, including keratoprostheses and tissue-engineered (TE) constructs as alternatives to donor corneas^4^,^5^,^6^,^7^,^8^. Nevertheless, most of these artificial corneas only partially satisfy the requirements, including biocompatibility, transparency, flexibility, and sufficient suturability strength. Ideal artificial corneas should be equivalent, and preferably superior to human cadaver corneas^9^.

Recently, decellularized corneas have received a great deal of attention as artificial corneal replacements, because they are usually completely free of cellular components and other immunogens from the corneal tissue through the use of chemical, biological, and physical methods, or combinations of these methods, thereby facilitating the suppression of the immune response to the tissue. However, these corneas still maintain the integrity of the extracellular matrix (ECM)^9^,^10^,^11^.

We have previously described the preparation of decellularized corneas using the high hydrostatic pressure (HHP) method^12^,^13^. The results of our *in vitro* studies comparing the efficacy of detergent versus HHP decellularization methods showed that the HHP method maintained corneal characteristics, such as the mechanical properties and glycosaminoglycan (GAG) content that are particularly important for the preservation of corneal structure, but not for transparency. Interestingly, subsequent *in vivo* studies, using interlamellar keratoplasty and deep anterior lamellar keratoplasty (DALK) in a xenotransplantation model, demonstrated that the decellularized cornea was opaque immediately after transplantation, but eventually became transparent within a follow-up period^14^. In general, corneal transparency is regulated by the specific architecture of the corneal stroma, which is a highly oriented, stacked collagen layer aligned in the vertical and horizontal directions^15^,^16^. With such a complex structure, the corneal stroma is very sensitive, and if it is irreversibly damaged by HHP decellularization, the transparency of the decellularized cornea may not recover. Therefore, we hypothesize that the properties of decellularized cornea contribute to the preservation of corneal ultrastructure even after the HHP decellularization. However, the ultrastructure of the decellularized cornea has yet to be demonstrated. In this study, we investigated the ultrastructural alterations of decellularized corneas transplanted into the rabbit stroma with different surgical procedures, using transmission electron microscopy (TEM).

## Results and Discussion

### Corneal decellularization

**Figure 1:**
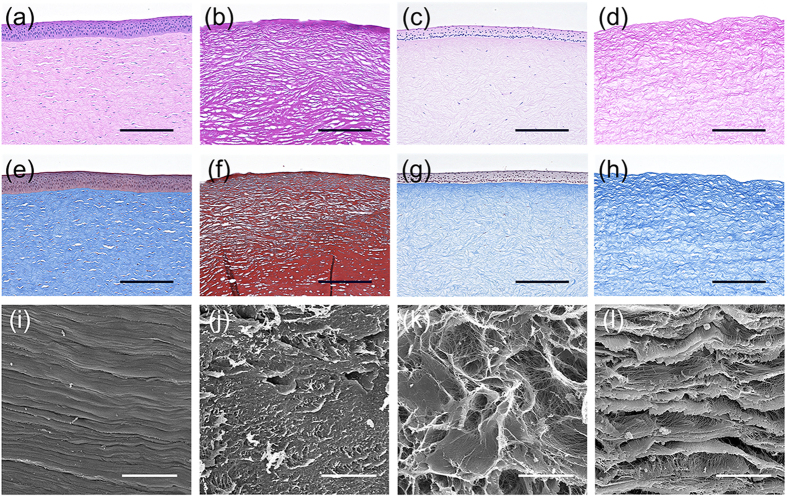
Histological and scanning electron microscopy (SEM) micrographs of the decellularized corneas. H&E and Masson’s trichrome staining of native cornea (**a,e**) and corneas treated with SDS (**b,f**), Triton X-100 (**c,g**), and high-hydrostatic pressure (HHP) (**d,h**). Histological analysis showing stratified epithelium and keratocytes in native cornea and no cell nuclei or debris in the decellularized cornea prepared by HHP. SEM observation of stroma in the native cornea (**i**) and corneas treated with SDS (**j**), Triton X-100 (**k**) and HHP (**l**) showing the similarity of lamella structure between native and decellularized cornea prepared by HHP. Scale bars: 100 μm in (**a–h**); 30 μm in (**i–l**).

**Figure 2:**
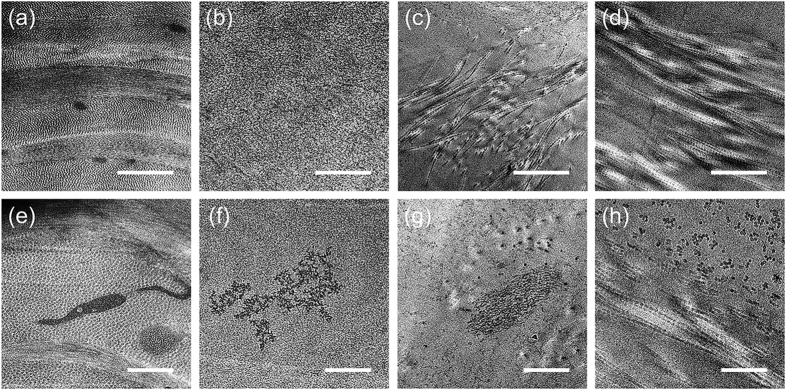
Transmission electron microscopy (TEM) micrographs of decellularized corneas obtained at different magnification. Low-magnified TEM images of the stroma in native cornea (**a**) and corneas treated with SDS (**b**), Triton X-100 (**c**) and HHP (**d**) showing orientations of collagen fibrils. Higher magnification focusing on the cell or cellular debris in the native cornea (**e**) and corneas treated with SDS (**f**), Triton X-100 (**g**) and HHP (**h**), indicating no cellular debris in the decellularized cornea prepared by HHP, which is correspond to the histological analysis. Scale bars: 1 μm in (**a–d**); 500 nm in (**e–h**).

Several decellularization techniques have been developed to prepare decellularized corneas^9^,^10^. Most of these procedures use chemical decellularization with detergents. We have performed a HHP decellularization procedure without detergent, and have demonstrated the efficacy of HHP decellularization for preparing decellularized corneas. However, the ultrastructure of the decellularized cornea, prepared with this procedure, remains to be determined. Here, we investigated the influence of decellularization on corneal ultrastructure. As controls, the decellularized corneas were prepared using the detergents, sodium dodecyl sulphate (SDS) or Triton X-100, both reported to be used for corneal decellularization^17^. The corneas subjected to SDS, Triton X-100, or HHP decellularization were primarily assessed by histological analyses and scanning electron microscopy (SEM) (Fig. 1). Histological analyses showed that almost all cells were eliminated in the cornea treated with SDS (Fig. 1b,f), while many keratocytes were present in the cornea treated with Triton X-100 (Fig. 1c,g). The lamellar structures of the corneas treated with these detergents were disarrayed. In contrast, the complete cell removal from the corneas was achieved by HHP decellularization (Fig. 1d,h), while the lamellar structures were relatively maintained when compared with the corneas treated with detergents. SEM also indicated that the corneas treated with detergents changed their aligned collagen fibril structures (Fig. 1j,k), while the corneas treated with HHP had hierarchical structures, which were composed of collagen fibrils arrayed regularly in parallel (Fig. 1l). These results were consistent with our histological data. Further analyses of the fibrillary arrangement of collagen within the decellularized corneas were conducted using TEM (Fig. 2). The native porcine cornea has a highly ordered, stacked collagen layer called the lamella (Fig. 2a), and keratocytes are present throughout the stroma (Fig. 2e). In the SDS-treated group, a loss of collagen fibrils and parallel loss of lamellar structure were observed (Fig. 2b). In the Triton X-100 treated group, the disorganization of oriented collagen fibrils resulted in an increased space between each collagen fibril (Fig. 2c). In addition, a disrupted cell shape was confirmed after either SDS or Triton X-100 detergent treatment (Fig. 2f,g). In contrast, the decellularized corneas prepared by HHP maintained collagen fibril lamellar structures, although some partially disorganized and oedematous collagen fibrils were observed (Fig. 2d,h).

These results may be due to the different cell removal mechanisms. SDS is an anionic detergent, which disrupts cellular membranes and denatures proteins by dissociating protein-protein interactions^18^. Consequently, the ultrastructure of collagen fibrils might be lost. Triton X-100 is a non-ionic detergent, which disrupts lipid-protein and lipid-lipid interactions, but does not disrupt protein-protein interactions^18^. Therefore, the ultrastructure of collagen fibrils might be maintained as shown in Fig. 2c. HHP decellularization does not disrupt protein-protein, protein-lipid, or lipid-lipid interactions, so that the ultrastructure of collagen fibrils is maintained.

### Circular dichroism (CD) analyses and differential scanning calorimetry (DSC) measurements

**Figure 3:**
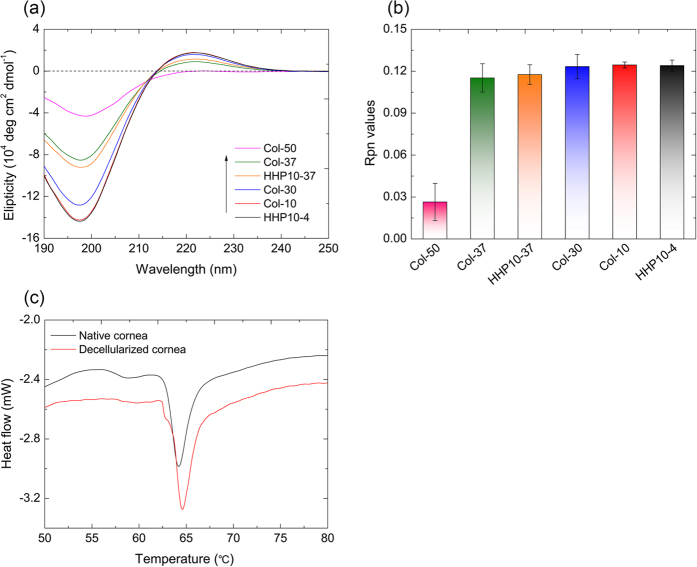
Circular dichroism (CD) spectroscopy and differential scanning calorimetry (DSC). CD spectra for collagen under various conditions (**a**) and Rpn values of collagen molecules estimated from CD spectra (**b**). Differential scanning calorimetry (DSC) analyses of the corneas treated with the high hydrostatic pressure (HHP) procedure (**c**). The Rpn value is expressed as the ratio of the positive peak intensity to the negative peak intensity.

CD is widely used for monitoring the conformational changes in proteins^19^. To characterize the impact of HHP treatment on the triple helical conformation of collagen, CD spectroscopy was conducted (Fig. 3a). The CD spectra of native collagen typically exhibited a positive peak around 220 nm, a negative peak around 197 nm, and a crossover point at about 210 nm, which is characteristic of the triple helical conformation^20^. To compare the thermal stability of collagen, collagen solutions were heated at 30 °C, 37 °C, and 50 °C. The positive and negative peaks decreased with increasing temperature. In particular, both peaks were significantly diminished at 50 °C. The collagen solution treated by HHP also showed a decrease in intensity of both peaks. To demonstrate whether the decrease of intensity was due to a conformational change of collagen fibrils, the Rpn values were estimated (Fig. 3b). The Rpn value is defined the ratio of the positive peak intensity to the negative peak intensity, and can be used to distinguish a triple helical conformation from a non-triple helical conformation^21^. Interestingly, the Rpn values for HHP treated collagen were comparable to that of native collagen, suggesting the triple helical conformation of HHP treated collagen was maintained, even though the intensity of both peaks was decreased.

Further characterization of the decellularized corneas was performed by DSC. The peak represents the temperature of maximum enthalpy (*T*_m_), which is caused by the destruction of a collagen structure in corneal tissues. The DSC spectra showed that the helix-coil transition of native porcine corneas and decellularized corneas occurred at around 64.1 °C and 64.6 °C, respectively (Fig. 3c). There was a slight difference of the helix-coil transition temperature between the native and decellularized corneas. These data were consistent with the previously reported *T*_m_ (64.8 ± 0.5) of porcine corneas^22^. Generally, HHP is hypothesized to cause the denaturation of proteins, and HHP treatment might, therefore, induce structural alterations in treated tissues. However, our results clearly showed that the triple helix structures of collagen fibrils and the collagen lamellar structures were maintained even after HHP treatment. These data suggest that by using our protocol for producing decellularized porcine cornea, complete decellularization was accomplished with less corneal stromal structural change.

### Interlamellar keratoplasty

**Figure 4:**
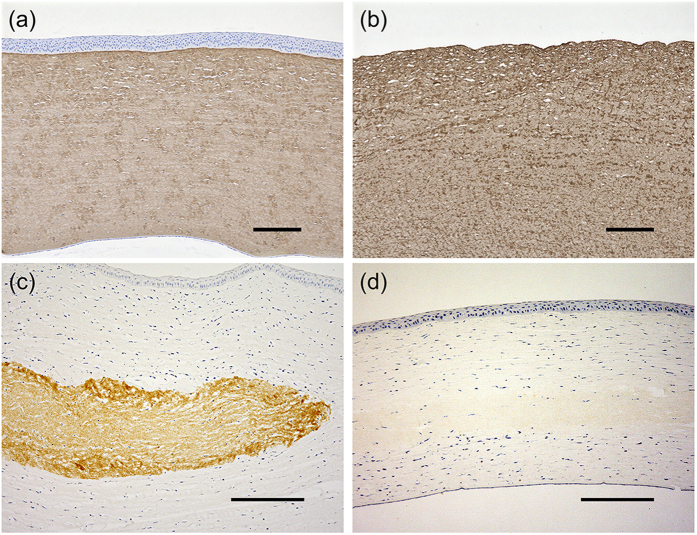
Immunohistological analysis of decellularized corneas. Native (**a**) and decellularized cornea (**b**) were immunostained with anti-type I collagen antibody. Representative images of type I collagen immunostaining of decellularized corneas at postoperative 2 weeks (**c**) and 8 weeks (**d**). Brown-stained areas indicate the transplanted decellularized corneas. Scale bars: 200 μm in (**a**,**b**); 250 μm in (**c,d).**

Light transmission is regulated by a highly organized extracellular matrix structure, which is very important for maintaining transparency^16^. Previous reports suggested that the decellularized cornea becomes transparent within 2 weeks after surgery, and the transparency is maintained for 12 months^13^. This phenomenon could be due to two possibilities. First, the opacification of the decellularized cornea could be caused by the swelling associated with the decellularization, even though the organized structure is maintained. According to the lattice theory, changes of the diameter of collagen fibrils and regular spacing between collagen fibrils can cause light scattering. In our study, the spacing of collagen fibrils in the decellularized cornea obviously expanded before surgery. We further confirmed that when the spacing of the collagen fibrils narrowed by dehydration, and using hypertonic solution, caused the opaque decellularized cornea to become transparent *in vitro*. This result suggested that the collagen structure of decellularized cornea was maintained, as shown in Fig. 3. Second, the transplanted decellularized cornea was remodelled by keratocytes derived from the rabbit. We analysed the residual decellularized corneas by immunohistochemistry using a monoclonal antibody that reacts with porcine collagen but not with rabbit collagen. Type I collagen was detected in decellularized corneal stroma, which is similar to the native cornea (Fig. 4a,b). At 2 weeks after surgery, collagen fibrils of the decellularized cornea were strongly stained with anti-type I collagen antibody, whereas at 8 weeks after surgery, staining of collagen fibrils was negligible (Fig. 4c,d). However, because we confirmed the structure of the decellularized cornea by histological analyses, the lack of immunoreactivity could be due to the destruction of the antigen epitopes necessary for antibody binding.

**Figure 5:**
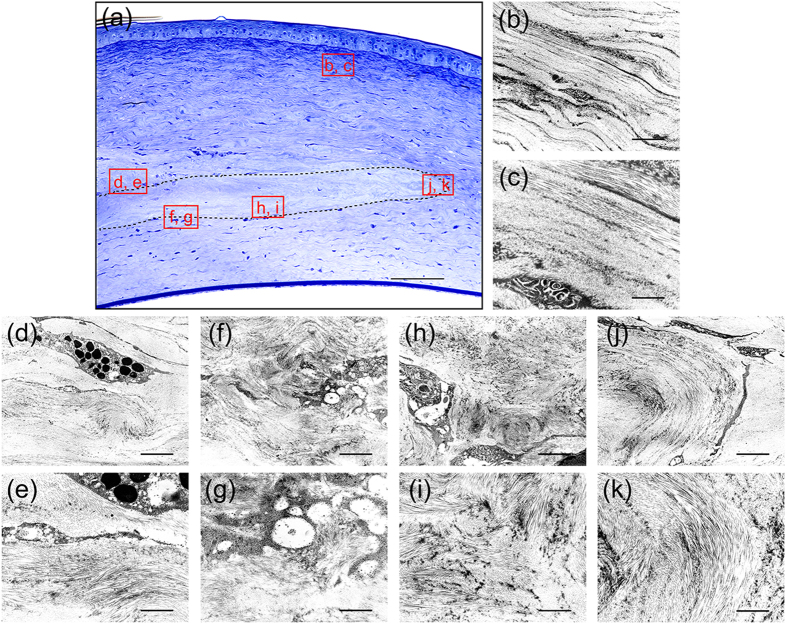
Structural analysis of decellularized corneas at 8 weeks after interlamellar keratoplasty. Optical micrograph of the decellularized cornea stained with toluidine blue at 8 weeks after interlamellar keratoplasty (**a**). The TEM images of the indicated squares in a are shown in (**b–k**). The dotted line region indicates the transplanted decellularized cornea. TEM image of receptive rabbit cornea showing two preferred orientations of collagen fibrils in successive lamella structure (**b,c**). Granulocyte was observed around the transplanted decellularized cornea (**d,e**). TEM observation revealed that the structure of the collagen fibrils was more randomly organized in the inside (**f– i**) than in the periphery (**j,k**) of the decellularized cornea. Scale bars: 100 μm in (**a**); 1 μm in (**b,d,f,h,j**); and, 3 μm in (**c,e,g,i,k**).

To verify these possible hypotheses, ultrastructural analyses of the transplanted decellularized corneas at 8 weeks after surgery were conducted (Fig. 5). TEM observation showed receptive rabbit cornea has preferred orientations of collagen fibrils in successive lamella structure (Fig. 5b,c), whereas the structure of the collagen fibrils in the transplanted decellularized cornea was more randomly organized in the inside than in the periphery, and the spacing of collagen fibrils was significantly narrowed. In addition, inside the decellularized corneas the lamellar structure also appeared randomly organized (Fig. 5f–i). However, in the periphery of the decellularized cornea, although the collagen fibrils were organized in a lamellar structure, similar to that of the native cornea. Nevertheless, few keratocytes infiltrated into the decellularized cornea (Fig. 5j,k), and granulocytes were still slightly observed around the transplanted decellularized cornea (Fig. 5d,e). Moreover, These results indicated that the decellularized cornea did not undergo remodelling, including the reproduction or reorganization of collagen shown at 8 weeks after surgery, and that a slight disorganization of collagen fibrils did not affect corneal transparency. Therefore, we hypothesize that the decrease of corneal transparency after decellularization is due to the swelling of the corneal stroma.

### Microkeratome-assisted anterior lamellar keratoplasty

**Figure 6:**
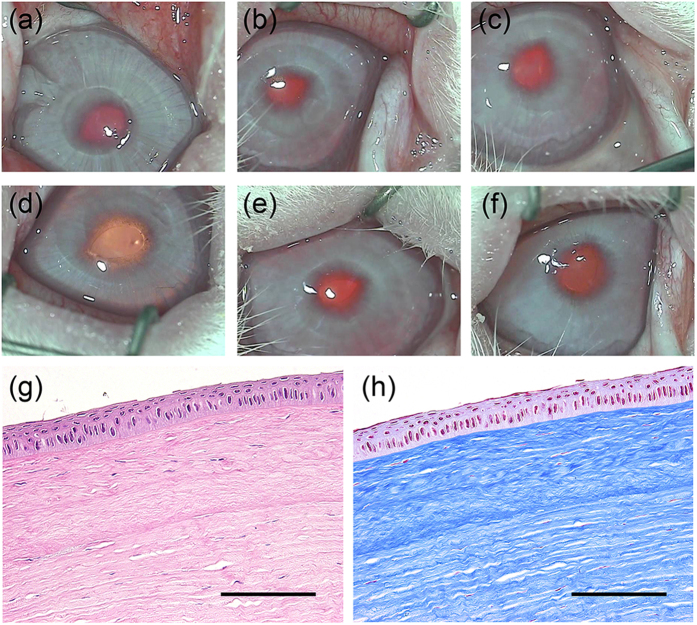
Representative macroscopic and histological images of decellularized cornea. Macroscopic images of the rabbit eye at 1 week (**a**), 2 weeks (**b**), 1 month (**c**), 2 months (**d**), 3 months (**e**), and 6 months (**f**) following the transplantation of decellularized cornea. H&E and Masson’s trichrome staining show transplanted decellularized cornea at 6 months after surgery. Scale bars: 2 mm in (**a–f**); 50 μm in (**g,h**).

**Figure 7:**
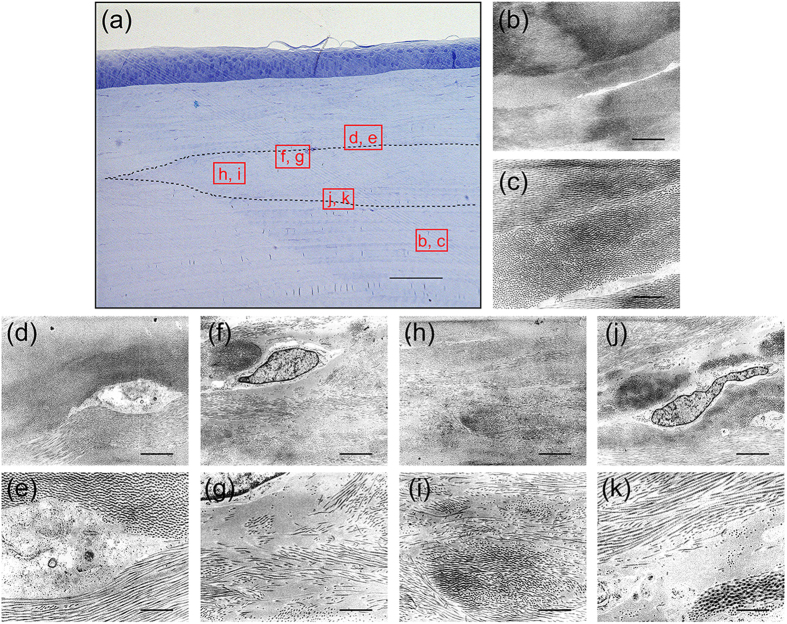
Structural analysis of decellularized corneas at 6 months after microkeratome-assisted anterior lamellar keratoplasty. Optical micrograph of the decellularized cornea stained with toluidine blue at 8 weeks after interlamellar keratoplasty (**a**). The TEM images of the indicated squares in A are shown in (**b–k**). The dotted line region indicates the transplanted decellularized cornea. TEM observation indicated that the collagen fibrils structure of the decellularized cornea was highly organized and similar to the host corneas (**d,e,h,i**). An irregular collagen arrangement and an increase of spacing between the collagen fibrils remained (**f,g,j,k**). Scale bars: 100 μm in (**a**); 1 μm in (**b,d,f,h,j**); and, 3 μm in (**c,e,g,i,k**).

To demonstrate the long-term *in vivo* behaviour of decellularized and transplanted corneas, a microkeratome-assisted anterior lamellar keratoplasty, which is used in clinical practice, was performed on six eyes of rabbits (Supplementary Fig. 2). The decellularized corneas became almost transparent at 1 week after surgery (Fig. 6a), and this occurred faster than with interlamellar keratoplasty. The transparency was maintained for at least 6 months after surgery (Fig. 6b–f). This was because the stromal bed produced by anterior keratectomy was prepared for placement of the decellularized cornea, so that the total thickness of the cornea was thinner using the microkeratome-assisted anterior lamellar keratoplasty than when using the interlamellar keratoplasty procedure. As a consequence, the decellularized cornea became less swollen faster than when using the interlamellar keratoplasty procedure. Histological analyses showed that the decellularized corneas had no inflammatory or immune responses to a host animal until 6 months after surgery (Fig. 6g,h). Finally, only a few cells derived from a rabbit could be recognized in the decellularized cornea (Fig. 6g,h and 7a). TEM indicated that the ultrastructure of the decellularized cornea was highly organized and similar to the host corneas (Fig. 7d,e,h,i). However, an irregular collagen arrangement and an increase of spacing between the collagen fibrils remained (Fig. 7f,g,j,k). The lattice theory suggests that the arrangement of the collagen fibrils forming a regular lattice structure is required, and that the structural array of collagen fibrils whose diameter is smaller than λ/4 will be transparent as they provide a pseudo-parallel array, and that there should be no substantial voids in the disordered lattice which are comparable in dimension to the wavelength of light^23^.

Our results suggest that the space between collagen fibrils in the decellularized cornea was smaller than λ/4. As a result, the transparency of decellularized corneas transplanted into rabbit corneas was maintained despite the different arrangement and spacing of collagen fibrils. These results were identical with those with interlamellar keratoplasty, and showed the remodelling of decellularized cornea did not occur at 6 months after surgery. Some studies showed that the inflammatory response in the interface of the corneal flap created by a microkeratome is extremely minimal, resulting in almost no healing response by the transformation of myofibroblasts, decomposition of damaged tissues, and the synthesis of a new extracellular matrix^24^. In another study, the remodelling of the decellularized cornea was possibly caused by growth factors secreted from the injured corneal epithelium. Therefore, wound healing responses are essential to remodel the decellularized cornea. The results of our study will provide new insight to further evaluate the use of decellularized corneas using animal models.

## Conclusions

We demonstrated that the decellularized corneas prepared by the HHP method were preserved in their lamella and collagen fibrils structure, when compared with the corneas prepared using the detergent method. The decellularized corneas were not remodelled with interlamellar keratoplasty and microkeratome-assisted anterior lamellar keratoplasty, while remaining stable in the rabbit cornea, and corneal transparency was also maintained for 6 months. The decellularized corneal procedure is promising for artificial cornea production.

## Materials and Methods

### Preparation of decellularized porcine cornea

Decellularized corneas were prepared as previously described^12^,^13^. Briefly, porcine eyes were purchased from a commercial slaughterhouse (Tokyo Shibaura Organ, Tokyo, Japan). Full-thickness corneas were taken from the whole eye using a surgical knife and washed with phosphate-buffered saline (PBS) containing 100 units/mL penicillin, 0.1 mg/mL streptomycin, and 3.5% w/v dextran (molecular weight: 70,000) (Tokyo Chemical Industry Co., Ltd., Tokyo, Japan). Detergent decellularization was done as follows. Corneas were immersed in a 1% w/v solution of either polyoxyethylene octylphenyl ether (Triton X-100) or sodium dodecyl sulphate (SDS) solution at 37 °C for 24 h, and then washed with PBS containing antibiotics for 24 h with shaking. HHP decellularization was done as follows. Corneas were placed into a cold isostatic pressure machine (Kobe Steel, Ltd., Kobe, Japan) and pressurized at 10,000 atm for 10 min at 10 °C. The pressurized corneas were then washed using EGM-2 medium (Lonza Ltd., Basel, Switzerland) containing 0.2 mg/mL DNase I, 100 units/mL penicillin, 0.1 mg/mL streptomycin, and 3.5% w/v dextran at 37 °C for 72 h.

### Histological analyses of decellularized corneas

Native and individual decellularized corneas were fixed with 4% paraformaldehyde for 24 h at 4 °C. Fixed corneas then underwent stepwise dehydration using ethanol, and were embedded in paraffin. Five μm serial sections were subjected to haematoxylin and eosin (H&E) and Masson’s trichrome (MT) staining and were observed using optical microscopy (BZ-X700, Keyence Corp., Tokyo, Japan).

### SEM analyses of decellularized corneas

Specimens were fixed with 2.5% glutaraldehyde (TAAB Laboratories Equipment, Ltd., Berks, UK) in PBS for 24 h at 4 °C. Fixed specimens were immersed in t-butyl alcohol after dehydration by a graded series of ethanol, and lyophilized. Then, the lyophilized specimens were coated with gold, and observed by SEM with accelerating voltage at 10 kV (S-3400NK, Hitachi High-Technologies Corp., Tokyo, Japan).

### TEM analyses of decellularized cornea

Additional corneal specimens were fixed with 2.5% glutaraldehyde in Sorense-Gomori’s PBS (pH 7.4) for 2 h at 4 °C. After being washed three times with PBS, specimens were post-fixed using 1% osmium tetroxide (Wako Pure Chemical Industries, Tokyo, Japan) for 2 h at 0 °C, dehydrated in ethanol, and embedded in epoxy resin. Ultrathin sections of 70 nm were cut using an ultra-microtome (Leica UC6/FC6, Leica Microsystems, Wetzlar, Germany) equipped with a diamond knife, and mounted on grids. Sections were observed by TEM with accelerating voltage at 200 kV (LEO 922 Omega, Carl Zeiss, Germany).

### Circular dichroism analyses

The triple helix structure of collagen after HHP treatment was characterized using a CD spectrometer (J-720 W, JASCO Corporation, Tokyo, Japan). Native collagen from bovine dermis (AteloCell^®^, Koken Co., Ltd., Tokyo, Japan) was diluted in water to a concentration of 3 × 10^−8^ M. The collagen solution was hydrostatically pressurized at 980 MPa at 10 °C for 10 min, and then kept at 4 °C (HHP10-4) or 37 °C (HHP10-37) for 72 h with shaking. As controls, the collagen solutions kept at 10 °C, 30 °C, 37 °C, and 50 °C for 72 h were used. These samples were measured five times each to obtain the average spectra. The Rpn value was expressed as a ratio of the positive peak intensity to the negative peak intensity and was calculated from the CD spectra.

### Differential scanning calorimetry

The thermal properties of the decellularized corneas were assessed by a DSC (DSC-8230D, Rigaku Corp., Tokyo, Japan). Briefly, tissue samples were hermetically sealed in an aluminum pan, and measured at a temperature range from 30 °C to 100 °C with a heating rate of 0.5 °C/min. A native cornea was used as a control sample.

### Animal transplantation model

Adult Japanese white rabbits (female, 2.5–3 kg, 12-weeks-old) (Kitayama Labes, Nagano, Japan) were used (n = 12). Animals were treated according to the ARVO Statement for the Use of Animals in Ophthalmic and Vision Research. All animal experiments were approved by the ethical committees for animal welfare of the National Institute for Materials Science (permission number: A9-2006-2) and Tokyo Medical and Dental University (permission number: 0080215), and approved methods for two kinds of animal experimentation are as follows.

Corneal sections of 160 μm thicknesses were prepared using a microkeratome (Moria 99 LSK Evolution 2; Moria SA, Antony, France), and were then decellularized by the HHP method as previously described. Animals were anaesthetised by the intravenous injection of 35 mg/kg sodium pentobarbital (Somnopentyl; Kyoritsu Seiyaku Corp., Tokyo, Japan) and topical 0.4% oxybuprocaine hydrochloride (Benoxil; Santen Pharmaceutical Co., Ltd., Osaka, Japan). The *in vivo* behaviour of the decellularized corneas were evaluated using two animal transplantation models, which are called the corneal pocket model and the corneal flap model (Supplementary Figs 1 and 2). Only one eye was operated on for each animal.

### Interlamellar keratoplasty

Decellularized corneal sections with thicknesses of 160 μm were trephined using a biopsy punch with a 2 mm diameter. The rabbit cornea was incised at the corneal stroma to about 50 μm in depth, and a corneal pocket of 6 mm in diameter was created with a crescent knife. The decellularized cornea was inserted into the corneal pocket. To prevent bacterial infection, topical 0.5% levofloxacin hydrate (Cravit; Daiichi Sankyo Co., Ltd., Tokyo, Japan) was administered in the eye until after three days of surgery. After 2 and 8 weeks, the rabbits were sacrificed by an overdose of sodium pentobarbital. The rabbit corneas, including the transplanted decellularized corneas, were removed and embedded in paraffin. Paraffin sections were immuno-stained with anti-human type I collagen antibody (Daiichi Fine Chemical Co., Ltd., Toyama, Japan).

### Microkeratome-assisted anterior lamellar keratoplasty

A corneal flap was prepared using a microkeratome with a custom-made hand piece for a rabbit cornea (MK-2000; Nidek Co., Ltd., Tokyo, Japan). A circular central wound of 4 mm in diameter and 200 μm in depth was prepared using a biopsy trephine (Kai Industries, Co., Ltd., Gifu, Japan) and surgical knife. Decellularized corneas with thicknesses of 160 μm were trephined using biopsy punches of 4 mm in diameter. Then, the decellularized cornea was placed on the corneal wound and covered with a corneal flap. To prevent the slippage of the transplanted decellularized cornea, a contact lens made from polyvinyl alcohol (PVA) was placed in the operated eye and the eyelid was sutured using 3-0 nylon sutures. Topical 0.5% levofloxacin hydrate (Cravit; Daiichi Sankyo Co., Ltd., Tokyo, Japan) was administered until the first week of surgery. Sutures were cut at 1 week after surgery. After 6 months, the rabbits were sacrificed with sodium pentobarbital and corneas, including the transplanted decellularized corneas, were removed. The tissues were evaluated by light microscopy and TEM using the same procedures described above.

## Additional Information

**How to cite this article**: Hashimoto, Y. *et al*. Ultrastructural analysis of the decellularized cornea after interlamellar keratoplasty and microkeratome-assisted anterior lamellar keratoplasty in a rabbit model. *Sci. Rep.* **6**, 27734; doi: 10.1038/srep27734 (2016).

## Supplementary Material

Supplementary Information
