# Supplementary material for: Ultrastructural analysis of the decellularized cornea after interlamellar keratoplasty and microkeratome-assisted anterior lamellar keratoplasty in a rabbit model
Source: Sci Rep. 2016 Jun 13;6:27734. doi: 10.1038/srep27734 (PMC4904214; doi:10.1038/srep27734)
Supplement: Supplementary Information [file srep27734-s1.pdf]

Supporting Information

Ultrastructural analysis of the decellularized cornea after interlamellar keratoplasty and microkeratome-assisted anterior lamellar keratoplasty in a rabbit model

Yoshihide Hashimoto, Shinya Hattori, Shuji Sasaki, Takako Honda, Tsuyoshi Kimura, Seiichi Funamoto, Hisatoshi Kobayashi & Akio Kishida

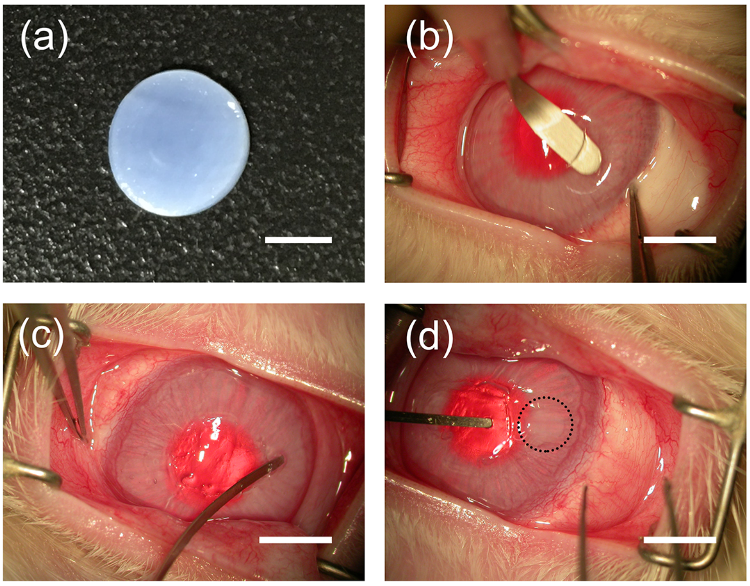

Supplementary Figure 1. The surgical procedure of interlamellar keratoplasty. Decellularized corneal disc of 2 mm in diameter (**a**). The corneal pocket was prepared using a crescent knife (**b**). The decellularized cornea was inserted into the corneal pocket (**c, d**). The dotted circle indicates the transplanted decellularized cornea. Scale bars: 1 mm in **a**; 4 mm in **b–d**.

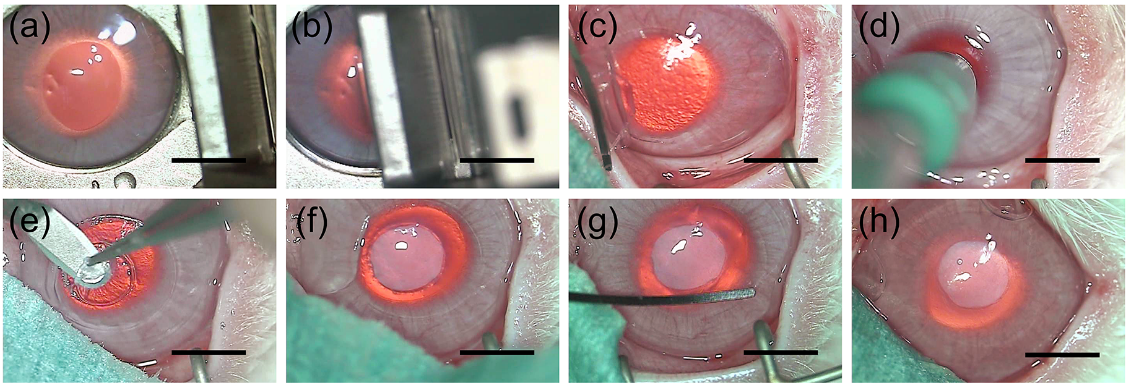

Supplementary Figure 2. The surgical procedure of microkeratome-assisted anterior lamellar keratoplasty. The corneal flap was prepared by a microkeratome (**A–C**). The recipient cornea was trephined using a 4.0 mm biopsy punch, and the discarded stromal tissue was removed (**D, E**). The decellularized cornea was placed on the corneal bed after keratectomy (**F**). The decellularized cornea was covered with a corneal flap, followed by a contact lens (**G, H**). Scale bar: 4 mm.
